# Outcome and risk factor of immune‐related adverse events and pneumonitis in patients with advanced or postoperative recurrent non‐small cell lung cancer treated with immune checkpoint inhibitors

**DOI:** 10.1111/1759-7714.13736

**Published:** 2020-11-17

**Authors:** Taisuke Isono, Naho Kagiyama, Kenji Takano, Chiaki Hosoda, Takashi Nishida, Eriko Kawate, Yoichi Kobayashi, Takashi Ishiguro, Youtaro Takaku, Kazuyoshi Kurashima, Tsutomu Yanagisawa, Noboru Takayanagi

**Affiliations:** ^1^ Department of Respiratory Medicine Saitama Cardiovascular and Respiratory Center Saitama Japan

**Keywords:** Immune checkpoint inhibitor, immune‐related adverse event, lung cancer, pneumonitis

## Abstract

**Background:**

Non‐small cell lung cancer (NSCLC) patients with pre‐existing respiratory diseases have been excluded in clinical trials of immune checkpoint inhibitor (ICI) therapy, and it is unknown whether the same degree of response can be expected as that in patients without pre‐existing respiratory diseases and if they are associated with increased risk for various immune‐related adverse events (irAEs) and ICI pneumonitis. This study aimed to evaluate predictive factors of clinical response, prognostic factors, risk factors of irAEs, and ICI pneumonitis in NSCLC patients with or without pre‐existing respiratory diseases.

**Methods:**

We conducted a retrospective study of 180 NSCLC patients who received ICI monotherapy of nivolumab, pembrolizumab, or atezolizumab from 1 January 2016 to 31 March 2019.

**Results:**

A total of 119 patients had pre‐existing respiratory diseases, including 20 with pre‐existing idiopathic interstitial pneumonias (IIPs). A total of 85 patients experienced irAEs, of which ICI pneumonitis was the most frequent adverse event, occurring in 27 patients. Of the three patients who died from irAEs, all from ICI pneumonitis, two had pulmonary emphysema and one had pre‐existing IIP. In multivariate analyses, irAEs were associated with objective response rate (ORR) and favorable OS, and IIPs were associated with increased risk for ICI pneumonitis. However, IIPs were not associated with low ORR or poor OS.

**Conclusions:**

Pre‐existing IIPs were a risk factor for ICI pneumonitis. However, this study showed that ICI therapy can be offered to patients with pre‐existing respiratory diseases with the expectation of the same degree of response as that in patients without pre‐existing respiratory diseases.

**Key points:**

Significant findings of the study: Pre‐existing IIPs were a risk factor for ICI pneumonitis, but objective response rate and prognosis of patients with IIPs were similar to those of other patients.

What this study adds: In patients with pre‐existing IIPs, ICI pneumonitis should be noted. However, ICI therapy can be offered to patients with pre‐existing respiratory diseases with the expectation of the same degree of response as that in patients without pre‐existing respiratory diseases

## Graphical abstract

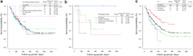

Pre‐existing IIPs were a risk factor for ICI pneumonitis. Objective response rate and prognosis of patients with IIPs were similar to those of other patients.

## Introduction

Immune checkpoint inhibitors (ICIs), including programmed cell death‐1 (PD‐1) inhibitor and programmed cell death ligand‐1 (PD‐L1) inhibitor, have become a standard treatment for patients with unresectable advanced or recurrent non‐small cell lung cancer (NSCLC). Nivolumab and pembrolizumab are PD‐1 inhibitors, and atezolizumab is a PD‐L1 inhibitor. In phase III trials, nivolumab, pembrolizumab, and atezolizumab as second‐line treatment provided longer overall survival (OS) than docetaxel in NSCLC patients.^1^, ^2^, ^3^, ^4^ Additionally, pembrolizumab as a first‐line treatment provided longer OS than platinum‐based chemotherapy in NSCLC patients with a PD‐L1 tumor proportion score (TPS) ≥50% and those with PD‐L1 TPS ≥1%.^5^, ^6^ Recently, phase III trials showed that combination therapy of ICIs and platinum‐based chemotherapy as first‐line treatment in NSCLC patients has a higher objective response rate (ORR) and offers longer progression‐free survival (PFS) and OS than chemotherapy alone, regardless of the PD‐L1 TPS.^7^, ^8^, ^9^ However, the clinical benefits remain limited to a subset of patients, and the predictive factors for response and prognosis in patients treated with ICIs are still unclear.

Additionally, ICIs can induce various immune‐related adverse events (irAEs). In phase III trials, irAEs developed in 20%–30% of patients.^3^, ^5^ In the clinical setting, irAEs developed more frequently than those in the phase III trials, with 30%–60% of patients affected.^10^, ^11^, ^12^ Nevertheless, knowledge of the frequency, risk factors, and management of irAEs in the clinical setting is insufficient. In particular, ICI‐related pneumonitis (ICI pneumonitis) accounts for 35% of anti‐PD‐1 inhibitor‐ and anti‐PD‐L1 inhibitor‐related deaths.^13^ Therefore, it is the most serious and life‐threatening irAE, as stated in the American Thoracic Society research statement published in 2019.^14^ In this statement, because patients with pre‐existing respiratory diseases were excluded in clinical trials, it is unknown whether such patients are associated with an increased risk for ICI pneumonitis.

Therefore, we retrospectively reviewed the clinical data of NSCLC patients treated with ICI monotherapy and aimed to identify predictive factors for response, prognosis, irAEs, and ICI pneumonitis in the clinical setting of these patients with or without pre‐existing respiratory diseases and those with idiopathic interstitial pneumonias (IIPs).

## Methods

### Subjects

From 1 January 2016 to 31 March 2019, 180 patients with unresectable advanced or recurrent NSCLC were treated with ICI monotherapy including nivolumab, pembrolizumab, and atezolizumab at our institution. The diagnosis of lung cancer was based on pathology or cytology findings. The clinical stage was established according to the eighth edition of the TNM classification. Information concerning tumorous characteristics including epidermal growth factor receptor (EGFR) mutation, anaplastic lymphoma kinase (ALK) rearrangement, c‐ros oncogene 1 (ROS‐1) rearrangement, BRAF V600E mutation, and PD‐L1 TPS was collected. The PD‐L1 TPS was assessed by means of the PD‐L1 immunohistochemistry 22C3 pharmDx assay. ICIs were administered until disease progression, intolerable toxicity, or patient refusal occurred. Pre‐existing respiratory diseases were diagnosed according to clinical features and high‐resolution computed tomography of the chest.

### Study design

We retrospectively investigated patients' background, ORR, OS, and development and management of irAEs, including ICI pneumonitis. We also investigated the predictive factors for ORR, OS, irAEs, and ICI pneumonitis. Clinical data were collected from medical records. Baseline clinical parameters were obtained within one month of the initial diagnosis. Pre‐existing respiratory diseases were divided into IIPs with or without pulmonary emphysema (PE), radiation‐induced pulmonary fibrosis with or without PE, PE without interstitial lung diseases (ILDs), and others. Radiographic patterns of IIPs were classified according to the international multidisciplinary classification of the IIPs and clinical practice guideline for the diagnosis of idiopathic pulmonary fibrosis.^15^, ^16^ Pulmonary emphysema was defined as focal areas or regions of low attenuation, usually without visible walls on chest CT.^17^ ORR was assessed according to the Response Evaluation Criteria in Solid Tumors (RECIST) version 1.1.^18^ OS was measured from first administration of the ICIs to death. The data cutoff date was 31 August 2019. The irAEs were assessed using the National Cancer Institute Common Terminology Criteria for Adverse Events (CTCAE) version 4.0. Radiographic patterns of ICI pneumonitis were classified into nonspecific interstitial pneumonia (NSIP) pattern, cryptogenic organizing pneumonia (COP) pattern, acute interstitial pneumonia/acute respiratory distress syndrome (AIP/ARDS) pattern, and hypersensitivity pneumonitis (HP) pattern.^19^ The NSIP pattern is ground‐glass opacities (GGOs) and reticular opacities predominantly in peripheral and lower lung distribution, traction bronchiectasis and lower lobe volume loss. The COP pattern is multifocal bilateral parenchymal consolidations, GGOs and reticular opacities with peripheral and lower lung distribution. The HP pattern is diffuse GGOs, centrilobular nodularities, and air trapping. The AIP/ARDS pattern is diffuse or multifocal GGOs or consolidations predominantly in dependent lung regions, lung volume loss and traction bronchiectasis.

This study was conducted in accordance with the Declaration of Helsinki and was approved by the institutional review board of Saitama Cardiovascular and Respiratory Center.

### Statistical analysis

Categorical data are summarized by frequency and percent, and continuous data are reported as the median and range. The Kaplan‐Meier method was used to estimate OS. Univariate and multivariate analyses were performed using a logistic regression model to determine predictors for ORR and a Cox proportional‐hazards model to determine predictors for OS, irAEs, and ICI pneumonitis. All statistical analyses were performed with EZR version 1.36 (Saitama Medical Center, Jichi Medical University, Saitama, Japan), which is a graphical user interface for R (The R Foundation for Statistical Computing, Vienna, Austria, version 3.4.3).^20^

## Results

### Patient characteristics

In total, 180 patients with advanced NSCLC underwent ICI monotherapy (Table 1). The median patient age was 68.5 (range, 40–83) years, 77.8% of the patients were male, 84.4% were smokers, 90.6% had an Eastern Cooperative Oncology Group performance status (ECOG PS) of 0 or 1, 33.9% had no pre‐existing respiratory diseases, 11.1% had IIPs, 11.7% had radiation‐induced pulmonary fibrosis, 41.1% had PE, 55.6% had adenocarcinoma, 78.9% were at stage IV, and 22.8% had brain metastasis. A total of 13 patients used immunosuppressants, and three patients had autoimmune diseases. A total of 21 patients had an *EGFR* mutation, none had ALK fusion, three patients had ROS1 fusion, and two patients had a *BRAF* mutation. The percentages of patients with PD‐L1 TPS <1%, 1%–49%, and ≥50% were 13.9%, 18.3%, and 32.8%, respectively. Among the patients, 11.1% had received molecular targeted therapy, 28.9% had received radiation therapy, and 18.3% were treated with ICIs as first‐line therapy. Of the 99 patients with PE, 74 did not have ILDs including IIPs or radiation‐induced pulmonary fibrosis. The median follow‐up period from initiation of ICIs was 299.5 (range: 9–1314) days, and the median number of treatment cycle of ICIs was four (range: 1–70). Patients treated with pembrolizumab had a higher frequency of PD‐L1 TPS ≥50% compared to those treated with nivolumab or atezolizumab. Most patients treated with atezolizumab had PD‐L1 TPS <1%. In addition, about half of the patients treated with pembrolizumab had received it as first‐line therapy.

**Table 1:** Characteristics of patients treated with immune checkpoint inhibitors (ICIs)

ICI	All (*n* = 180)	Nivolumab (*n* = 99)	Pembrolizumab (*n* = 70)	Atezolizumab (*n* = 11)
Age at ICI initiation	68.5 (40–83)	68.0 (40–83)	70.0 (44–83)	65.0 (49–80)
Sex, male	140 (77.8)	79 (79.8)	55 (78.6)	6 (54.5)
Smoker	152 (84.4)	84 (84.8)	59 (84.3)	9 (81.8)
ECOG PS 0 or 1	163 (90.6)	89 (89.9)	64 (91.4)	10 (90.9)
Pre‐existing respiratory disease				
PE	99 (55.0)	57 (57.6)	38 (54.3)	4 (36.4)
RIPF	21 (11.7)	15 (15.2)	4 (5.7)	2 (18.2)
IIPs	20 (11.1)	12 (12.1)	8 (11.4)	0 (0.0)
UIP pattern	3 (1.7)	1 (1.0)	2 (2.9)	0 (0.0)
Probable UIP pattern	6 (3.3)	4 (4.0)	2 (2.9)	0 (0.0)
Indeterminate for UIP pattern	9 (5.0)	5 (5.1)	4 (5.7)	0 (0.0)
NSIP pattern	2 (1.1)	2 (2.0)	0 (0.0)	0 (0.0)
Asthma	8 (4.4)	3 (3.0)	5 (7.1)	0 (0.0)
Old tuberculosis	3 (1.7)	1 (1.0)	2 (2.9)	0 (0.0)
MAC infection	1 (0.6)	1 (1.0)	0 (0.0)	0 (0.0)
Bronchiectasis	1 (0.6)	1 (1.0)	0 (0.0)	0 (0.0)
Silicosis	1 (0.6)	0 (0.0)	1 (1.4)	0 (0.0)
Autoimmune disease				
Chronic thyroiditis	2 (1.1)	0 (0.0)	1 (1.4)	1 (9.1)
PBC	1 (0.6)	1 (1.0)	0 (0.0)	0 (0.0)
Use of corticosteroid or immunosuppressant	13 (7.2)	9 (9.1)	4 (5.7)	0 (0.0)
Histological type				
Adenocarcinoma	100 (55.6)	54 (54.5)	37 (52.9)	9 (81.8)
Squamous cell carcinoma	47 (26.1)	28 (28.3)	19 (27.1)	0 (0.0)
Pleomorphic carcinoma	4 (2.2)	1 (1.0)	3 (4.3)	0 (0.0)
Adenosquamous carcinoma	2 (1.1)	2 (2.0)	0 (0.0)	0 (0.0)
LCNEC	1 (0.6)	0 (0.0)	1 (1.4)	0 (0.0)
NOS	26 (14.4)	14 (14.1)	10 (14.3)	2 (18.2)
*EGFR* mutation				
Exon 19 deletion	11 (6.1)	6 (6.1)	4 (5.7)	1 (9.1)
L858R	7 (3.9)	4 (4.0)	3 (4.3)	0 (0.0)
Minor mutation	3 (1.7)	3 (3.0)	0 (0.0)	0 (0.0)
−	130 (72.2)	64 (64.6)	56 (80.0)	10 (90.9)
NA	29 (16.1)	22 (22.2)	7 (10.0)	0 (0.0)
ALK rearrangement				
+	0 (0.0)	0 (0.0)	0 (0.0)	0 (0.0)
−	139 (77.2)	70 (70.7)	59 (84.3)	10 (90.9)
NA	41 (22.8)	29 (29.3)	11 (15.7)	1 (9.1)
ROS‐1 rearrangement				
+	3 (1.7)	0 (0.0)	3 (4.3)	0 (0.0)
−	79 (43.9)	32 (32.3)	38 (54.3)	9 (81.8)
NA	98 (54.4)	67 (67.7)	29 (41.4)	2 (18.2)
BRAF V600E mutation				
+	2 (1.1)	1 (1.0)	1 (1.4)	0 (0.0)
−	31 (17.2)	15 (15.2)	11 (15.7)	5 (45.5)
NA	147 (81.7)	83 (83.8)	58 (82.9)	6 (54.5)
PD‐L1 TPS				
<1%	25 (13.9)	15 (15.2)	2 (2.9)	8 (72.7)
1–49%	43 (23.9)	17 (17.2)	13 (32.9)	3 (27.3)
≥50%	49 (27.2)	4 (4.0)	45 (64.3)	0 (0.0)
NA	63 (35.0)	63 (63.6)	0 (0.0)	0 (0.0)
Stage				
III	38 (21.1)	21 (21.2)	15 (21.4)	2 (18.2)
IV	142 (78.9)	78 (78.8)	55 (78.6)	9 (81.8)
Brain metastasis	41 (22.8)	21 (21.2)	15 (21.4)	5 (45.5)
Prior treatment for brain metastasis	33 (18.3)	17 (17.2)	12 (17.1)	4 (36.4)
Prior molecular targeted therapy	20 (11.1)	12 (12.1)	7 (10.0)	1 (9.1)
EGFR‐TKI	18 (10.0)	11 (11.1)	6 (8.6)	1 (9.1)
Prior radiotherapy	52 (28.9)	33 (33.3)	13 (32.9)	6 (54.4)
Prior thoracic radiotherapy	33 (18.3)	22 (22.2)	7 (10.0)	4 (36.4)
Line of ICI therapy				
First‐line	33 (18.3)	0 (0.0)	33 (47.1)	0 (0.0)
Second‐line	66 (36.7)	37 (37.4)	26 (37.1)	3 (27.3)
≥Third‐line	81 (45.0)	62 (62.6)	11 (15.7)	8 (72.7)
Number of ICI therapies	4 (1–70)	3 (1–70)	5.5 (1–33)	4 (1–11)
Follow‐up period (days)	299.5 (9–1314)	242 (9–1314)	362 (11–856)	233 (62–456)

Data are presented as *n*, median (range) or *n* (%).

ALK, anaplastic lymphoma kinase; ECOG PS, Eastern Cooperative Oncology Group performance status; EGFR, epidermal growth factor receptor; ICIs, immune checkpoint inhibitors; IIPs, idiopathic interstitial pneumonias; LCNEC, large‐cell neuroendocrine carcinoma; MAC, *Mycobacterium avium* complex; NA, not available; NOS, not otherwise specified; NSIP, nonspecific interstitial pneumonia; PBC, primary biliary cirrhosis; PD‐L1, programmed cell death ligand‐1; PE, pulmonary emphysema; RIPF, radiation‐induced pulmonary fibrosis; ROS‐1, c‐ros oncogene 1; TKI, tyrosine kinase inhibitor; TPS, tumor proportion score; UIP, usual interstitial pneumonia.

### IrAEs profile

**Figure 1:**
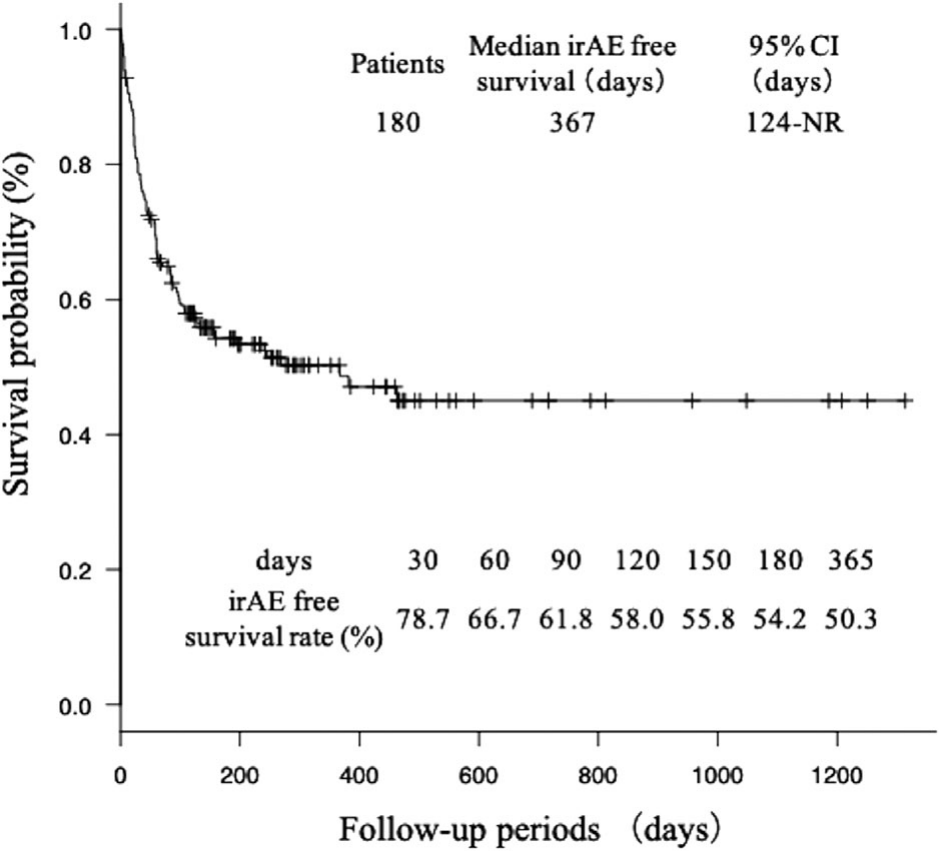
Kaplan‐Meier curves showing irAE free survival and irAE free survival rate at 30 days, 60 days, 90 days, 120 days, 150 days, 180 days and 365 days. CI, confidence interval; IIPs, idiopathic interstitial pneumonias; ILD, interstitial lung disease; irAE, immune‐related adverse event; NR, not reached; NSIP, nonspecific interstitial pneumonia; PE, pulmonary emphysema; UIP, usual interstitial pneumonia.

Of the 180 patients treated with ICIs, 121 (67.2%) developed adverse events, and the most common of these other than irAEs were drug‐related fever and bacterial pneumonia (Table 2). IrAEs were observed in 85 (47.2%) patients, including 27 (15.0%) with ICI pneumonitis, 24 (13.3%) with rash, 23 (12.8%) with thyroid dysfunction, 20 (11.1%) with diarrhea or colitis, 13 (7.2%) with hepatitis, five (2.8%) with nephritis, four (2.2%) with arthritis, and three (1.7%) with isolated adrenocorticotropic hormone deficiency. A total of 21 (11.7%) patients experienced irAEs of grade 3 or higher in which ICI pneumonitis was the most frequent adverse event. Systemic corticosteroids were administered to 36 (42.4%) patients. Among the 34 patients requiring discontinuation of ICIs, seven (20.6%) underwent retreatment with ICIs and two experienced recurrence of irAEs. Most patients who develop side effects develop them within one year, especially within 90 days (Fig 1). In patients treated with nivolumab, pembrolizumab, and atezolizumab, 45 (45.5%), 38 (54.3%), and two (18.2%) had irAEs, and 14 (14.1%), 12 (17.1%), and 1 (9.1%) had ICI pneumonitis, respectively.

**Table 2:** Adverse events including immune‐related adverse events (irAEs)

Events	Any grade	Grade ≥3	Corticosteroid treatment	Retreatment with ICIs	irAEs after retreatment
Any AEs including irAEs	121 (67.2)	24 (13.3)			
Drug‐related fever	26 (14.4)	1 (0.6)			
Pneumonia	12 (6.7)	10 (5.6)			
Asthma	4 (2.2)	0 (0.0)			
Allergic rhinitis	3 (1.7)	0 (0.0)			
Infusion reaction	1 (0.6)	0 (0.0)			
LTBI	1 (0.6)	0 (0.0)			
Pyothorax	1 (0.6)	1 (0.6)			
Choledocholithic cholangitis	1 (0.6)	1 (0.6)			
Any irAEs	85 (47.2)	21 (11.7)	36 (42.4)	7 (20.6)	2 (28.6)
ICI pneumonitis	27 (15.0)	10 (5.6)	20 (74.1)	1 (5.6)	0 (0.0)
Rash	24 (13.3)	2 (1.1)	4 (16.7)	1 (50.0)	1 (100.0)
Thyroid dysfunction	23 (12.8)	0 (0.0)	0 (0.0)	1 (20.0)	0 (0.0)
Colitis or diarrhea	20 (11.1)	2 (1.1)	6 (30.0)	3 (60.0)	1 (33.3)
Hepatitis	13 (7.2)	3 (1.7)	2 (15.4)	0 (0.0)	NA
Nephritis	5 (2.8)	0 (0.0)	1 (20.0)	NA	NA
Arthritis	4 (2.2)	0 (0.0)	1 (25.0)	1 (100.0)	0 (0.0)
Isolated ACTH deficiency	3 (1.7)	3 (1.7)	0 (0.0)	NA	NA
Myocarditis	1 (0.6)	1 (0.6)	1 (100.0)	0 (0.0)	NA
Uveitis	1 (0.6)	0 (0.0)	0 (0.0)	NA	NA
Eosinophilic fasciitis	1 (0.6)	1 (0.6)	1 (100.0)	0 (0.0)	NA

Data are presented as *n*, median (range) or *n* (%).

ACTH, adrenocorticotropic hormone; AEs, adverse events; ICIs, immune checkpoint inhibitors; irAEs, immune‐related adverse events; LTBI, latent tuberculosis infection; NA, not available.

### Predictive factors of antitumor response to ICIs

Of the 180 patients treated with ICIs, complete response was achieved in four patients (2.2%) and partial response in 44 (24.4%). Stable disease was present in 51 (28.3%) patients, and progressive disease occurred in 81 (45.0%). The overall ORR was 26.7%. The ORR of patients treated with nivolumab, pembrolizumab, and atezolizumab were 19.2%, 40.0%, and 9.1%, respectively. The ORR of patients with no pre‐existing respiratory disease, IIPs, radiation‐induced pulmonary fibrosis, and PE were 19.7%, 35.0%, 19.0%, and 31.1%, respectively. Univariate analysis indicated that type of ICIs, PD‐L1, line of ICI therapy, eosinophil count, lymphocyte count, lactate dehydrogenase (LDH) level, neutrophil‐to‐lymphocyte ratio (NLR), eosinophil count after treatment with ICIs, and irAEs were factors associated with antitumor response to ICIs (Table S1). In a multivariate logistic regression model, only LDH level and irAEs were significantly associated with antitumor response to ICIs (Table 3).

**Table 3:** Multivariate analyses of objective response rate and prognostic factors of all‐cause mortality in patients treated with immune checkpoint inhibitors (ICIs)

Analyses of objective response rate		*n*	ORR (%)	OR (95% CI)	*P*‐value
PD‐L1 TPS	<1%	25	12.0	Reference	
	1–49%	43	16.3	1.270 (0.229–7. 300)	0.785
	≥50%	49	51.0	5.140 (0.836–31.600)	0.077
	NA	63	20.6	2.200 (0.403–12.000)	0.363
ICIs	Nivolumab	99	19.2	Reference	
	Atezolizumab	11	9.1	0.917 (0.074–11.300)	0.946
	Pembrolizumab	70	40.0	1.850 (0.495–6.950)	0.360
Line of ICI therapy	First‐line	33	48.5	0.876 (0.205–3.74)	0.858
	Second‐line	66	19.7	Reference	
	≥Third‐line	81	23.5	1.960 (0.725–5.320)	0.184
Eosinophils (/μL)	<500	158	22.8	Reference	
	≥500	22	54.5	2.190 (0.618–7.750)	0.225
Lymphocytes (/μL)	<1500	103	20.4	Reference	
	≥1500	77	35.1	1.310 (0.545–3.150)	0.547
LDH (U/L)	≥230	68	16.2	Reference	
	<230	112	33.0	3.270 (1.340–8.020)	0.009
NLR	≥5	51	15.7	Reference	
	<5	129	31.0	2.940 (0.969–8.910)	0.057
Eosinophils after starting ICIs (/μL)	<500	123	18.7	Reference	
	≥500	57	43.9	1.990 (0800–4.960)	0.139
irAEs	None	95	15.8	Reference	
	Present	85	38.8	2.460 (1.070–5.650)	0.034
Analyses of prognostic factors		*n*	OS(days)	HR (95% CI)	*P*‐value
ECOG PS	0–1	163	468	Reference	
	2–3	17	123	3.499 (1.756–6.969)	< 0.001
PD‐L1 TPS	≥50%	49	NR	Reference	
	1–49%	43	444	1.778 (0.713–4.435)	0.217
	<1%	25	272	1.980 (0.685–5.720)	0.207
	NA	63	315	1.183 (0.430–3.253)	0.745
Stage	III	38	NR	Reference	
	IV	142	367	1.867 (1.025–3.400)	0.041
ICIs	Pembrolizumab	70	NR	Reference	
	Nivolumab	99	296	2.493 (1.123–5.536)	0.025
	Atezolizumab	11	307	2.803 (0.938–8.371)	0.065
Line of ICI therapy	First‐line	33	NR	Reference	
	Second‐line	66	289	1.134 (0.414–3.105)	0.807
	≥Third‐line	81	385	0.692 (0.243–1.968)	0.490
WBC (/μL)	<9000	146	467	Reference	
	≥9000	34	359	1.876 (0.985–3.570)	0.056
Monocytes (/μL)	<600	116	592	Reference	
	≥600	64	296	1.170 (0.680–2.014)	0.570
Lymphocytes (/μL)	≥1500	77	592	Reference	
	<1500	103	296	1.313 (0.748–2.303)	0.343
LDH (U/L)	<230	112	604	Reference	
	≥230	68	315	1.370 (0.888–2.112)	0.154
NLR	<5	129	493	Reference	
	≥5	51	281	0.848 (0.446–1.614)	0.615
LMR	≥3	83	744	Reference	
	<3	97	281	1.782 (0.985–3.222)	0.056
PLR	<300	139	472	Reference	
	≥300	41	226	1.711 (0.966–3.030)	0.066
Eosinophils after starting ICIs (/μL)	≥500	57	744	Reference	
	<500	123	322	1.191 (0.711–1.997)	0.507
irAEs	Present	85	670	Reference	
	None	95	303	1.637 (1.041–2.573)	0.033

CI, confidence interval; ECOG PS, Eastern Cooperative Oncology Group performance status; HR, hazard ratio; ICIs, immune checkpoint inhibitors; irAEs, immune‐related adverse events; LDH, lactate dehydrogenase; LMR, lymphocyte‐to‐monocyte ratio; NA, not available; NLR, neutrophil‐to‐lymphocyte ratio; OR, odds ratio; ORR, objective response rate; PD‐L1, programmed cell death ligand‐1; PLR, platelet‐to‐lymphocyte ratio; TPS, tumor proportion score; WBC, white blood cell.

### Prognostic factors of all‐cause mortality in patients treated with ICIs

**Figure 2:**
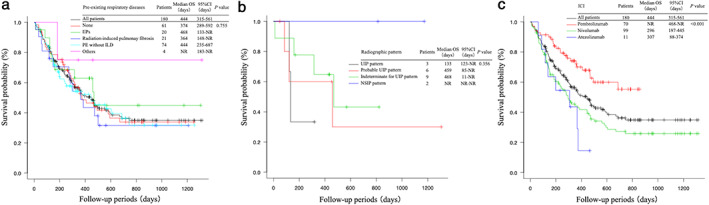
Kaplan‐Meier curves showing (**a**) surOS stratified by pre‐existing respiratory diseases; (**b**) OS stratified by radiographic pattern of IIPs; and (**c**) OS stratified by type of ICI in non‐small cell lung cancer patients treated with immune checkpoint inhibitors. The log‐rank test of the difference between survival curves of patients with and without pre‐existing respiratory disease was not significant. On the other hand, the log‐rank test revealed a significant survival benefit in patients treated with pembrolizumab compared to those treated with nivolumab or atezolizumab. CI, confidence interval; IIPs, idiopathic interstitial pneumonias; ILD, interstitial lung disease; NR, not reached; NSIP, nonspecific interstitial pneumonia; PE, pulmonary emphysema; UIP, usual interstitial pneumonia.

The median OS was 444 days (95% confidence interval [CI]: 315–561) in all patients treated with ICIs (Fig 2). Univariate analysis indicated that ECOG PS, stage, type of ICI, PD‐L1, line of ICI therapy, white blood cell (WBC) count, monocyte count, lymphocyte count, LDH level, NLR, lymphocyte‐to‐monocyte ratio, platelet‐to‐lymphocyte ratio (PLR), eosinophil count after treatment with ICIs, and irAEs were prognostic factors (Table S2). In a multivariate Cox proportional hazard model, ECOG PS, type of ICI, stage IV, and irAEs were independent prognostic factors of all‐cause mortality (Table 3). Kaplan‐Meier curves for OS stratified by pre‐existing respiratory diseases, including IIPs, revealed no significant differences in patient prognosis between the various diseases (Fig 2a). Patients with IIPs of NSIP pattern tended to have a longer OS and patients with IIPs of UIP pattern tended to have a shorter OS (Fig 2b). However, the number of patients in each group was very small and there was no significant difference in prognosis. Other respiratory diseases included bronchial asthma in three and stable pulmonary tuberculosis in one. There were only four cases, two with PD‐L1 ≥50% and one with unknown PD‐L1, which may be due to the longest survival in this study. On the other hand, stratified by type of ICI revealed that patients treated with pembrolizumab had significantly longer median OS than those treated with nivolumab or atezolizumab (Fig 2c).

### Risk factors for irAEs

Univariate analysis indicated that age, WBC count, and lymphocyte count were risk factors for irAEs (Table S3). In a multivariate Cox proportional hazard model, only age and lymphocyte count were risk factors for irAEs (Table 4).

**Table 4:** Univariate and multivariate analyses of immune‐related adverse events (irAEs) and pneumonitis

Analyses of irAEs		*n*	irAEs (%)	HR (95% CI)	*P*‐value
Age	≥75	42	31.0	Reference	
	<75	138	52.2	2.109 (1.167–3.813)	0.013
WBC (/μL)	<9000	146	43.8	Reference	
	≥9000	34	61.8	1.649 (0.991–2.743)	0.054
Lymphocytes (/μL)	<1500	103	37.9	Reference	
	≥1500	77	59.7	1.553 (1.001–2.409)	0.049
Analyses of pneumonitis		*n*	Pneumonitis (%)	HR (95% CI)	*P*‐value
Pre‐existing respiratory disease	None	61	6.6	Reference	
	IIPs	20	35.0	4.350 (1.225–15.440)	0.023
	RIPF	21	19.0	3.096 (0.735–13.040)	0.124
	PE without ILD	74	16.2	2.088 (0.645–6.760)	0.219
	Others	4	0.0	<0.001 (0.000–Inf)	0.998
PD‐L1 TPS	<1%	49	24.0	3.897 (0.911–16.670)	0.067
	1–49%	43	3.0	Reference	
	≥50%	25	23.7	2.488 (0.660–9.380)	0.178
	NA	63	9.5	1.480 (0.352–6.222)	0.593
WBC (/μL)	<9000	146	12.3	Reference	
	≥9000	34	26.5	1.263 (0.492–3.243)	0.627
Eosinophils (/μL)	<500	158	12.7	Reference	
	≥500	22	31.8	1.853 (0.705–4.873)	0.211
Monocytes (/μL)	<600	116	8.6	Reference	
	≥600	64	26.6	2.080 (0.875–4.941)	0.097
Albumin (g/dL)	≥4	50	6.0	Reference	
	<4	126	19.0	2.090 (0.588–7.420)	0.254
	NA	4	0.0	<0.001 (0.000–Inf)	0.998
CRP (mg/dL)	<1	96	7.3	Reference	
	≥1	84	23.8	1.711 (0.645–4.537)	0.281

CI, confidence interval; CRP, C‐reactive protein; HR, hazard ratio; ICIs, immune checkpoint inhibitors; IIPs, idiopathic interstitial pneumonias; ILD, interstitial lung disease; irAEs, immune‐related adverse events; NA. not available; PD‐L1, programmed cell death ligand‐1; PE, pulmonary emphysema; RIPF, radiation‐induced pulmonary fibrosis; TPS, tumor proportion score; WBC, white blood cell.

### Risk factors for ICI pneumonitis

Univariate analysis indicated that age, IIPs, PD‐L1, WBC count, eosinophil count, monocyte count, and albumin and C‐reactive protein (CRP) levels were risk factors for ICI pneumonitis (Table S4). In a multivariate Cox proportional hazard model, however, IIPs were the only risk factor for ICI pneumonitis (Table 4).

### Characteristics of ICI pneumonitis

**Figure 3:**
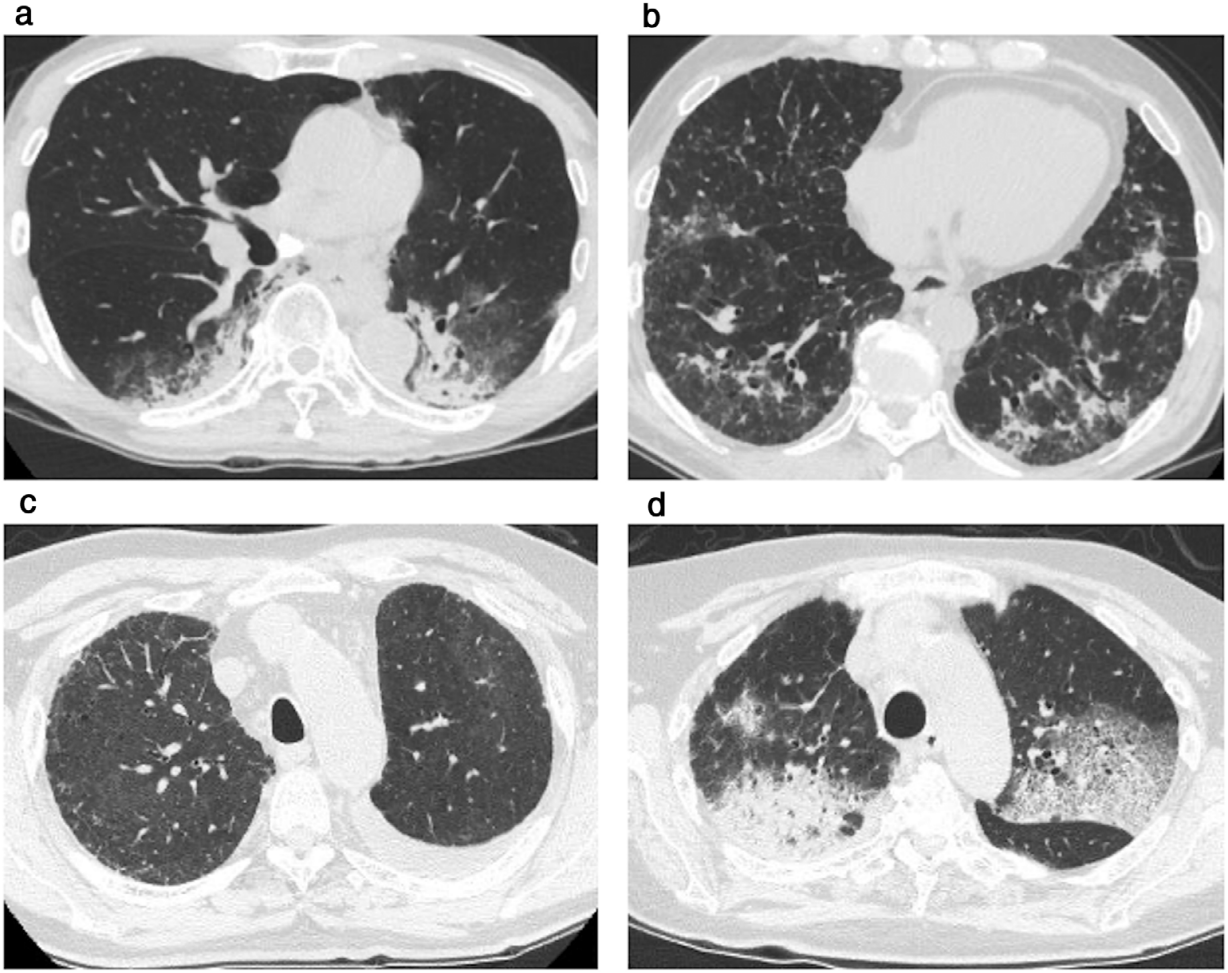
Radiographic pattern of immune checkpoint inhibitor (ICI)‐related pneumonitis (ICI pneumonitis. (**a**) COP pattern; (**b**) NSIP pattern; (**c**) HP pattern; and (**d**) AIP/ARDS pattern. COP, cryptogenic organizing pneumonia; NSIP, nonspecific interstitial pneumonia; HP, hypersensitivity pneumonitis; AIP/ARDS, acute interstitial pneumonia/acute respiratory distress syndrome.

**Figure 4:**
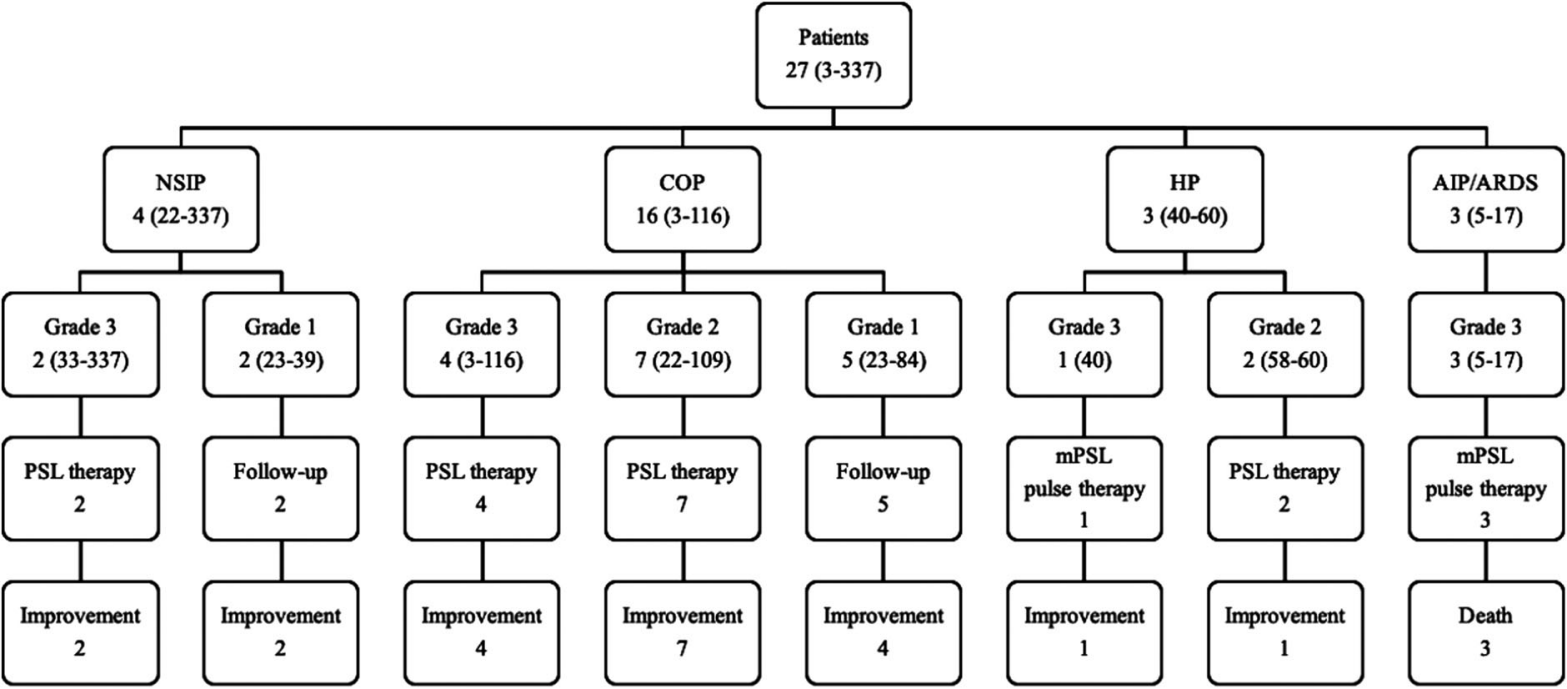
Radiographic pattern, grade, treatment, and outcome of immune checkpoint inhibitor (ICI)‐related pneumonitis (ICI pneumonitis). Data are presented as number of patients or range of time in days to onset of ICI pneumonitis. AIP/ARDS, acute interstitial pneumonia/acute respiratory distress syndrome; COP, cryptogenic organizing pneumonia; HP, hypersensitivity pneumonitis; mPSL, methylprednisolone; NSIP, nonspecific interstitial pneumonia; PSL, prednisolone.

Of the 27 patients with ICI pneumonitis, the most common radiographic pattern was the COP pattern (16 patients; Fig 3a) followed by NSIP pattern (four patients; Fig 3b), HP pattern (three patients; Fig 3c), and AIP/ARDS pattern (three patients; Fig 3d). Time to onset of ICI pneumonitis with AIP/ARDS pattern ranged from five to 17 days and tended to be shorter than that of ICI pneumonitis with other radiographic patterns (Fig 4). Among the three patients who developed ICI pneumonitis with AIP/ARDS pattern, all three had respiratory diseases other than lung cancer (two with pulmonary emphysema and one with IIP), all three were at grade 3 severity at the onset of ICI pneumonitis, and all three died. All of the patients with ICI pneumonitis of grade 2 or higher were treated with corticosteroids, whereas all of the patients with ICI pneumonitis of grade 1 were observed without treatment.

## Discussion

In this study, we revealed predictive factors for clinical outcome and irAEs in patients with advanced NSCLC treated with ICI monotherapy in a clinical setting. Predictive factors for clinical response were LDH level, and irAEs. Predictive factors for prognosis were ECOG PS, stage, type of ICI, and irAEs. Pembrolizumab had the highest frequency of irAEs and the best tumor response and prognosis. About half of the patients experienced irAEs, the risk factors for which were age and lymphocyte count. The most frequent irAE was ICI pneumonitis, and all three deaths were due to ICI pneumonitis with an AIP/ARDS radiographic pattern. Although IIPs were a significant risk factor for ICI pneumonitis, there were no significant differences in the ORR and OS between patients with IIPs and those without respiratory diseases.

Previously, it was reported that several factors predict the response and prognosis in patients treated with ICIs. In phase III trials, PD‐L1 expression was associated with OS in NSCLC patients treated with ICIs.^2^, ^3^ Tamiya *et al*. showed that ECOG PS ≥2, liver metastasis, and lung metastasis were predictive of poor PFS in NSCLC patients treated with nivolumab.^21^ Additionally, several studies reported that irAEs were associated with clinical response and prognosis. Sato *et al*.^10^ and Toi *et al*.^22^ respectively investigated 38 and 70 NSCLC patients treated with nivolumab and reported that patients with irAEs had significantly higher ORR than those without irAEs (63.6 vs. 7.4% and 57 vs. 12%, respectively). Haratani *et al*.^23^ investigated 134 NSCLC patients treated with nivolumab and reported that the patients with irAEs had significantly longer median OS than those without irAEs (not reached vs. 11.1 months). Similarly, Ricciuti *et al*.^24^ studied 195 NSCLC patients treated with nivolumab and reported that the patients with irAEs experienced significantly longer median OS than those without irAEs (17.8 vs. 4.0 months), and patients who developed ≥2 irAEs had significantly longer median OS than those with one or no irAEs (26.8 vs. 11.9 vs. 4.0 months). The present study also revealed that irAEs were associated with both ORR and OS in NSCLC patients treated with ICIs. In contrast, Ksienski *et al*.^25^ studied 271 patients treated with nivolumab or pembrolizumab and showed that treatment interruption due to irAEs was associated with a lower median OS than was continuous treatment (8.27 vs. 14.54 months). Therefore, appropriate assessment and management of irAEs is necessary.

Several studies have shown risk factors of irAEs. Diehl *et al*.^11^ reported that baseline lymphocyte and eosinophil counts were associated with irAEs in solid tumor patients treated with ICIs. A pooled analysis including NSCLC patients from four trials of ICIs showed that patients aged ≥75 years had a lower incidence of grade 3 or 4 adverse events than patients aged <65 years (23 vs. 47%).^26^ However, because a pooled analysis including NSCLC patients from three trials for pembrolizumab showed that there were no differences in the incidence of irAEs between patients aged <75 and ≥75 years (24.8 vs. 25.0%),^27^ it remains controversial whether age is related to the incidence of irAEs.

In the present study, most of the patients who developed ICI pneumonitis or liver injury after ICI therapy discontinued ICIs permanently. According to the American Society of Clinical Oncology clinical practice guideline, if patients develop irAEs, ICI therapy is continued with close monitoring for grade 1 irAEs, is held for grade 2 or 3 irAEs until they improve to grade 1 or less, and is permanently discontinued for grade 4 irAEs except endocrinopathies.^28^ Patients with grade 3 or 4 ICI pneumonitis and liver injury were required to permanently discontinue ICI therapy. Mouri *et al*.^29^ reported the clinical differences between patients who discontinued ICIs and those who retreated after occurrences of irAEs. They found that patients who discontinued ICIs tended to more frequently have ICI pneumonitis, thyroid dysfunction, and liver injury than those retreated from therapy.

Although several clinical trials revealed that 2.5% to 5% of patients developed ICI pneumonitis,^14^ its incidence was higher in the clinical setting than in the clinical trials, and 5.4% to 16.9% of patients experienced ICI pneumonitis.^10^, ^11^, ^30^ Tone *et al*.^31^ reported that patients with ICI pneumonitis of grade 3 or higher were associated with shorter median OS than those with ICI pneumonitis of grade 2 or lower or no ICI pneumonitis. A retrospective study reported that radiographic patterns were associated with grades of ICI pneumonitis, with the AIP/ARDS pattern associated with the highest grade, followed by the COP pattern, and the NSIP and HP patterns associated with lower grades.^32^ Several studies have reported risk factors of ICI pneumonitis. Cui *et al*.^33^ revealed that prior radiotherapy and combination therapy, defined as treatment with anti‐PD‐1 antibody and chemotherapy, targeted therapy, or anticytotoxic T‐lymphocyte‐associated antigen‐4 antibody, were significantly associated with ICI pneumonitis in a multivariable logistic regression model. Oshima *et al*.^34^ analyzed the Food and Drug Administration Adverse Event Reporting System database and investigated the association between pneumonitis and the combination of nivolumab and EGFR‐tyrosine kinase inhibitor (TKI). They reported that 18 of the 70 patients who were treated with the combination developed pneumonitis (25.7%), with the order of treatment in 15 patients identified as EGFR‐TKI after nivolumab administration. A systematic review and meta‐analysis showed that the incidence of ICI pneumonitis in NSCLC was higher than that in melanoma.^35^ Additionally, a retrospective study showed the incidence in NSCLC of the adenocarcinoma histological pattern to be lower than that in NSCLC of the squamous histological pattern.^36^ Several studies showed the efficacy and safety of ICIs in patients with pre‐existing ILD or interstitial lung abnormalities, which are defined as areas of increased lung density on lung computed tomography in individuals with no known ILD.^30^ Kanai *et al*.^37^ investigated 216 NSCLC patients who had received nivolumab and reported that the incidence of ICI pneumonitis was significantly higher in patients with pre‐existing ILD than in patients without ILD (31 vs. 12%). There were no significant differences in the ORR (27 vs.13%) and median PFS (2.7 vs. 2.9 months). Nakanishi *et al*.^30^ studied 83 NSCLC patients who had received nivolumab or pembrolizumab and found that the patients with ICI pneumonitis had a significantly higher frequency of interstitial lung abnormalities than those without ICI pneumonitis (42.9 vs. 10.1%).There were no significant differences in the response to the ICIs. Fujimoto *et al*.^38^ studied the efficacy and safety of nivolumab for NSCLC patients with mild IIPs. They reported that two of the 18 patients (11.1%) with IIPs developed ICI pneumonitis. The ORR was 39%, median PFS was 7.4 months, and median OS was 15.6 months. Similar to the previous studies, the incidence of ICI pneumonitis in the present study was significantly higher in patients with pre‐existing IIPs than in those without pre‐existing respiratory diseases (35.0 vs. 6.6%), and the ORR in the patients with IIPs was 35.0%. In addition, patients with IIPs tended to have a longer OS, although the difference was not significant. In this study, patients treated with atezolizumab had the poorest ORR and OS, and none of the patients with IIP received atezolizumab. Furthermore, although IIPs was a risk factor for the development of ICI pneumonitis in this study, two‐thirds of ICI‐pneumonitis patients were Grade 1–2, with a fatality rate of only 10%, and patients with irAEs had better OS than those without irAEs. These findings may have contributed to the present study.

This study has several limitations. First, because it was retrospective, some patient characteristics were not available. Second, it was performed at a single hospital, and only Japanese patients were treated. Third, the sample size was small. Finally, diagnoses of ICI pneumonitis were largely based on clinical course and CT findings. Only a small percentage of patients underwent bronchoalveolar lavage to exclude pneumonia. However, pneumonitis was not resolved by antimicrobial drugs.

In summary, the incidence of irAEs might be a useful predictor of clinical response and prognosis in NSCLC patients treated with ICIs, and we believe that appropriate management of irAEs can lead to clinical benefit. Because all three patient deaths were due to ICI pneumonitis, we consider ICI pneumonitis to be the most important irAE, and radiological pattern classification was useful for predicting the prognosis of ICI pneumonitis. Pre‐existing IIPs were a risk factor for ICI pneumonitis; however, this study showed that ICI therapy can be offered to patients with pre‐existing respiratory diseases with the expectation of the same degree of response as that in patients without pre‐existing respiratory diseases.

## Disclosure

The authors declare there are no conflicts of interest.

## Supporting information


**Table S1** Univariate and multivariate analyses of objective response rate.
**Table S2** Univariate and multivariate analyses of prognostic factors of all‐cause mortality in patients treated with ICIs.
**Table S3** Univariate and multivariate analyses of irAEs.
**Table S4** Univariate and multivariate analyses of ICI pneumonitis.
