# Supplementary material for: Outcome and risk factor of immune‐related adverse events and pneumonitis in patients with advanced or postoperative recurrent non‐small cell lung cancer treated with immune checkpoint inhibitors
Source: Thorac Cancer. 2020 Nov 17;12(2):153–64. doi: 10.1111/1759-7714.13736 (PMC7812074; doi:10.1111/1759-7714.13736)
Supplement: Supplementary file 1 — Table S1 Univariate and multivariate analyses of objective response rate. Table S2 Univariate and multivariate analyses of prognostic factors of all‐cause mortality in patients treated with ICIs. Table S3 Univariate and multivariate analyses of irAEs. Table S4 Univariate and multivariate analyses of ICI pneumonitis. [file TCA-12-153-s001.docx]

| **Table S1** Univariate and multivariate analyses of objective response rate | | | | | |
| --- | --- | --- | --- | --- | --- |
| Parameter | | n | ORR  (%) | Univariate |  |
|  |  |  |  | OR (95% CI) | *P* value |
| Age (years) | ≥ 75 | 42 | 23.8 | Reference |  |
|  | < 75 | 138 | 27.5 | 1.22 (0.545–2.710) | 0.633 |
| Sex | Female | 40 | 17.5 | Reference |  |
|  | Male | 140 | 29.3 | 1.950 (0.799–4.770) | 0.142 |
| Smoking history | Non-smoker | 28 | 17.9 | Reference |  |
|  | Smoker | 152 | 28.3 | 1.810 (0.648–5.080) | 0.257 |
| ECOG PS | 2–3 | 17 | 11.8 | Reference |  |
|  | 0–1 | 163 | 28.2 | 2.950 (0.649–13.400) |  |
| Pre-existing respiratory disease | None | 61 | 19.7 | Reference |  |
|  | IIPs | 20 | 35.0 | 2.200 (0.721–6.700) | 0.166 |
|  | RIPF | 21 | 19.0 | 0.961 (0.273–3.380) | 0.950 |
|  | PE without ILD | 74 | 31.1 | 1.840 (0.827–4.100) | 0.135 |
|  | Others | 4 | 50.0 | 4.080 (0.521–32.000) | 0.181 |
| Histologic type | Adenocarcinoma | 100 | 24.0 | Reference |  |
|  | SCC | 47 | 25.5 | 1.090 (0.488–2.420) | 0.840 |
|  | Others | 33 | 36.4 | 1.810 (0.777–4.210) | 0.169 |
| EGFR mutation | Negative | 130 | 27.7 | Reference |  |
|  | Positive | 21 | 28.6 | 1.040 (0.376–2.900) | 0.934 |
|  | NA | 29 | 20.7 | 0.681 (0.256–1.810) | 0.441 |
| PD-L1 TPS | < 1% | 25 | 12.0 | Reference |  |
|  | 1–49% | 43 | 16.3 | 1.770 (0.415–7.530) | 0.441 |
|  | ≥ 50% | 49 | 51.0 | 8.680 (2.310–32.600) | 0.001 |
|  | NA | 63 | 20.6 | 2.170 (0.565–8.310) | 0.260 |
| Stage | Ⅳ | 142 | 24.6 | Reference |  |
|  | Ⅲ | 38 | 34.2 | 1.590 (0.735–3.440) | 0.239 |
| Prior molecular targeted therapy | None | 160 | 26.3 | Reference |  |
|  | Present | 20 | 30.0 | 1.200 (0.435–3.340) | 0.721 |
| Prior radiotherapy | Other than thorax | 19 | 15.8 | Reference |  |
|  | None | 128 | 28.9 | 2.170 (0.596–7.890) | 0.240 |
|  | Thorax | 33 | 24.2 | 1.710 (0.393–7.410) | 0.475 |
| ICIs | Nivolumab | 99 | 19.2 | Reference |  |
|  | Atezolizumab | 11 | 9.1 | 0.421 (0.051–3.490) | 0.423 |
|  | Pembrolizumab | 70 | 40.0 | 2.810 (1.410–5.610) | 0.003 |
| Line of ICI therapy | 1st-line | 33 | 48.5 | 3.840 (1.540–9.560) | 0.004 |
|  | 2nd-line | 66 | 19.7 | Reference |  |
|  | ≥ 3rd-line | 81 | 23.5 | 1.250 (0.564–2.770) | 0.583 |
| WBC (/μL) | ≥ 9000 | 34 | 23.5 | Reference |  |
|  | < 9000 | 146 | 27.4 | 1.230 (0.513–2.930) | 0.646 |
| Platelets (/μL) | < 300000 | 123 | 24.4 | Reference |  |
|  | ≥ 300000 | 57 | 31.6 | 1.430 (0.715–2.860) | 0.312 |
| Neutrophils (/μL) | < 6000 | 132 | 26.5 | Reference |  |
|  | ≥ 6000 | 48 | 27.1 | 1.030 (0.489–2.170) | 0.939 |
| Eosinophils (/μL) | < 500 | 158 | 22.8 | Reference |  |
|  | ≥ 500 | 22 | 54.5 | 4.070 (1.620–10.200) | 0.003 |
| Basophils (/μL) | < 100 | 114 | 23.7 | Reference |  |
|  | ≥ 100 | 66 | 31.8 | 1.500 (0.766)) | 0.236 |
| Monocytes (/μL) | ≥ 600 | 64 | 23.4 | Reference |  |
|  | < 600 | 116 | 28.4 | 1.300 (0.642–2.630) | 0.467 |
| Lymphocytes (/μL) | < 1500 | 103 | 20.4 | Reference |  |
|  | ≥ 1500 | 77 | 35.1 | 2.110 (1.080–4.120)) | 0.029 |
| LDH (U/L) | ≥ 230 | 68 | 16.2 | Reference |  |
|  | < 230 | 112 | 33.0 | 2.560 (1.200–5.440) | 0.015 |
| Albumin (g/dL) | < 4 | 126 | 26.2 | Reference |  |
|  | ≥ 4 | 50 | 28.0 | 1.100 (0.526–2.280) | 0.807 |
|  | NA | 4 | 25.0 | 0.939 (0.094–9.530) | 0.957 |
| CRP (mg/dL) | < 1 | 96 | 21.9 | Reference |  |
|  | ≥ 1 | 84 | 32.1 | 1.690 (0.869–3.290) | 0.122 |
| NLR | ≥ 5 | 51 | 15.7 | Reference |  |
|  | < 5 | 129 | 31.0 | 2.420 (1.040–5.610) | 0.040 |
| LMR | < 3 | 97 | 21.6 | Reference |  |
|  | ≥ 3 | 83 | 32.5 | 1.740 (0.896–3.400) | 0.102 |
| PLR | ≥ 300 | 41 | 17.1 | Reference |  |
|  | < 300 | 139 | 29.5 | 2.030 (0.833–4.960) | 0.119 |
| Eosinophils after starting ICIs (/μL) | < 500 | 123 | 18.7 | Reference |  |
|  | ≥ 500 | 57 | 43.9 | 3.400 (1.700–6.790) | < 0.001 |
| irAEs | None | 95 | 15.8 | Reference |  |
|  | Present | 85 | 38.8 | 3.380 (1.680–6.840) | < 0.001 |
| ICI pneumonitis | None | 153 | 24.2 | Reference |  |
|  | Present | 27 | 40.7 | 2.160 (0.919–5.050) | 0.077 |
| ORR, objective response rate; OR, odds ratio; CI, confidence interval; ECOG PS, Eastern Cooperative Oncology Group performance status; IIPs, RIPF, radiation-induced pulmonary fibrosis; idiopathic interstitial pneumonias; PE, pulmonary emphysema; ILD, interstitial lung disease; SCC, squamous cell carcinoma; EGFR, epidermal growth factor receptor; NA, not available; PD-L1, programmed cell death ligand-1; TPS, tumor proportion score; ICIs, immune checkpoint inhibitors; WBC, white blood cell; LDH, lactate dehydrogenase; CRP, C-reactive protein; NLR, neutrophil-to-lymphocyte ratio; LMR, lymphocyte-to-monocyte ratio; PLR, platelet-to-lymphocyte ratio; irAEs, immune-related adverse events. | | | | | |

| **Table S2** Univariate and multivariate analyses of prognostic factors of all-cause mortality in patients treated with ICIs | | | | | |
| --- | --- | --- | --- | --- | --- |
| Parameter | | n | OS  (days) | Univariate |  |
|  |  |  |  | HR (95% CI) | *P* value |
| Age (years) | < 75 | 138 | 385 | Reference |  |
|  | ≥ 75 | 42 | 445 | 0.968 (0.597–1.569) | 0.894 |
| Sex | Female | 40 | 408 | Reference |  |
|  | Male | 140 | 445 | 1.172 (0.710–1.934) | 0.535 |
| Smoking history | Non-smoker | 28 | 580 | Reference |  |
|  | Smoker | 152 | 359 | 1.473 (0.805–2.695) | 0.209 |
| ECOG PS | 0–1 | 163 | 468 | Reference |  |
|  | 2–3 | 17 | 123 | 3.26 (1.952–6.371) | < 0.001 |
| Pre-existing respiratory disease | None | 61 | 374 | Reference |  |
|  | IIPs | 20 | 468 | 0.805 (0.387–1.676) | 0.562 |
|  | RIPF | 21 | 364 | 1.169 (0.618–2.211) | 0.631 |
|  | PE without ILD | 74 | 444 | 1.053 (0.668–1.658) | 0.825 |
|  | Others | 4 | NR | 0.374 (0.051–2.730) | 0.332 |
| Histologic type | Adenocarcinoma | 100 | 459 | Reference |  |
|  | SCC | 47 | 359 | 1.023 (0.637–1.642) | 0.925 |
|  | Others | 33 | 472 | 0.888 (0.516–1.528) | 0.668 |
| EGFR mutation | Positive | 21 | 472 | Reference |  |
|  | Negative | 130 | 444 | 1.009 (0.535–1.906) | 0.977 |
|  | NA | 29 | 296 | 1.050 (0.487–2.265) | 0.901 |
| PD-L1 TPS | ≥ 50% | 49 | NR | Reference |  |
|  | 1–49% | 43 | 444 | 2.626 (1.383–4.985) | 0.003 |
|  | < 1% | 25 | 272 | 3.794 (1.942–7.411) | < 0.001 |
|  | NA | 63 | 315 | 2.596 (1.452–4.640) | 0.001 |
| Stage | Ⅲ | 38 | NR | Reference |  |
|  | Ⅳ | 142 | 367 | 1.904 (1.097–3.304) | 0.022 |
| Prior molecular targeted therapy | Present | 20 | 580 | Reference |  |
|  | None | 160 | 408 | 1.109 (0.576–2.134) | 0.757 |
| Prior radiotherapy | None | 128 | 467 | Reference |  |
|  | Thorax | 33 | 364 | 1.123 (0.668–1.887) | 0.661 |
|  | Other than thorax | 19 | 307 | 1.270 (0.671–2.401) | 0.463 |
| ICIs | Pembrolizumab | 70 | NR | Reference |  |
|  | Nivolumab | 99 | 296 | 2.272 (1.160–3.772) | < 0.001 |
|  | Atezolizumab | 11 | 307 | 3.192 (1.426–7.145) | 0.005 |
| Line of ICI therapy | 1st-line | 33 | NR | Reference |  |
|  | 2nd-line | 66 | 289 | 2.272 (1.160–4.453) | 0.017 |
|  | ≥ 3rd-line | 81 | 385 | 2.029 (1.055–3.904) | 0.034 |
| WBC (/μL) | < 9000 | 146 | 467 | Reference |  |
|  | ≥ 9000 | 34 | 359 | 1.661 (1.041–2.651) | 0.033 |
| Platelets (/μL) | < 300000 | 123 | 467 | Reference |  |
|  | ≥ 300000 | 57 | 296 | 1.249 (0.821–1.900) | 0.299 |
| Neutrophils (/μL) | < 6000 | 132 | 468 | Reference |  |
|  | ≥ 6000 | 48 | 364 | 1.459 (0.941–2.265) | 0.092 |
| Eosinophils (/μL) | ≥ 500 | 22 | 472 | Reference |  |
|  | < 500 | 158 | 408 | 1.203 (0.657–2.203) | 0.550 |
| Basophils (/μL) | ≥ 100 | 66 | 467 | Reference |  |
|  | < 100 | 114 | 374 | 1.089 (0.720–1.647) | 0.686 |
| Monocytes (/μL) | < 600 | 116 | 592 | Reference |  |
|  | ≥ 600 | 64 | 296 | 1.730 (1.155–2.590) | 0.008 |
| Lymphocytes (/μL) | ≥ 1500 | 77 | 592 | Reference |  |
|  | < 1500 | 103 | 296 | 1.639 (1.087–2.471) | 0.018 |
| LDH (U/L) | < 230 | 112 | 604 | Reference |  |
|  | ≥ 230 | 68 | 315 | 1.769 (1.189–2.632) | 0.005 |
| Albumin (g/dL) | ≥ 4 | 50 | 592 | Reference |  |
|  | < 4 | 126 | 352 | 1.522 (0.959–2.416) | 0.074 |
|  | NA | 4 | NR | < 0.001 (0.000–NA) | 0.995 |
| CRP (mg/dL) | < 1 | 96 | 472 | Reference |  |
|  | ≥ 1 | 84 | 359 | 1.241 (0.835–1.844) | 0.286 |
| NLR | < 5 | 129 | 493 | Reference |  |
|  | ≥ 5 | 51 | 281 | 1.917 (1.257–2.924) | 0.003 |
| LMR | ≥ 3 | 83 | 744 | Reference |  |
|  | < 3 | 97 | 281 | 2.523 (1.653–3.850) | < 0.001 |
| PLR | < 300 | 139 | 472 | Reference |  |
|  | ≥ 300 | 41 | 226 | 1.826 (1.167–2.856) | 0.008 |
| Eosinophils after starting ICIs (/μL) | ≥ 500 | 57 | 744 | Reference |  |
|  | < 500 | 123 | 322 | 1.772 (1.122–2.798) | 0.014 |
| irAEs | Present | 85 | 670 | Reference |  |
|  | None | 95 | 303 | 1.805 (1.200–2.716) | 0.005 |
| ICI pneumonitis | None | 153 | 408 | Reference |  |
|  | Present | 27 | 472 | 1.032 (0.604–1.764) | 0.908 |
| ICIs, immune checkpoint inhibitors; OS, overall survival; HR, hazard ratio; CI, confidence interval; ECOG PS, Eastern Cooperative Oncology Group performance status; IIPs, idiopathic interstitial pneumonias; RIPF, radiation-induced pulmonary fibrosis; PE, pulmonary emphysema; ILD, interstitial lung disease; SCC, squamous cell carcinoma; EGFR, epidermal growth factor receptor; NA, not available; PD-L1, programmed cell death ligand-1; TPS, tumor proportion score; NR, not reached; WBC, white blood cell; LDH, lactate dehydrogenase; CRP, C-reactive protein; NLR, neutrophil-to-lymphocyte ratio; LMR, lymphocyte-to-monocyte ratio; PLR, platelet-to-lymphocyte ratio; irAEs, immune-related adverse events. | | | | | |

| **Table S3** Univariate and multivariate analyses of irAEs | | | | | |
| --- | --- | --- | --- | --- | --- |
| Parameter | | n | irAEs  (%) | Univariate |  |
|  |  |  |  | HR (95% CI) | *P* value |
| Age | ≥ 75 | 42 | 31.0 | Reference |  |
|  | < 75 | 138 | 52.2 | 2.139 (1.183–3.865) | 0.012 |
| Sex | Female | 140 | 47.1 | Reference |  |
|  | Male | 40 | 47.5 | 0.915 (0.549–1.525) | 0.734 |
| Smoking history | Non-smoker | 28 | 39.3 | Reference |  |
|  | Smoker | 152 | 48.7 | 1.296 (0.687–2.443) | 0.424 |
| ECOG PS | 0–1 | 163 | 46.6 | Reference |  |
|  | 2–3 | 17 | 52.9 | 1.978 (0.9863.968) | 0.055 |
| Pre-existing respiratory disease | None | 61 | 41.0 | Reference |  |
|  | IIPs | 20 | 65.0 | 1.885 (0.962–3.692) | 0.065 |
|  | RIPF | 21 | 47.6 | 1.263 (0.606–2.631) | 0.534 |
|  | PE without ILD | 74 | 48.6 | 1.192 (0.715–1.988) | 0.501 |
|  | Others | 4 | 25.0 | 0.544 (0.074–4.023) | 0.551 |
| Histologic type | SCC | 47 | 44.7 | Reference |  |
|  | Adenocarcinoma | 100 | 48.0 | 1.115 (0.667–1.862) | 0.679 |
|  | Others | 33 | 48.5 | 1.067 (0.557–2.045) | 0.846 |
| EGFR mutation | Positive | 21 | 28.6 | Reference |  |
|  | Negative | 130 | 56.2 | 2.249 (0.978–5.173) | 0.056 |
|  | NA | 29 | 20.7 | 0.626 (0.202–1.941) | 0.417 |
| PD-L1 TPS | < 1% | 25 | 32.0 | Reference |  |
|  | 1–49% | 43 | 36.4 | 1.153 (0.523–2.541) | 0.725 |
|  | ≥ 50% | 49 | 59.3 | 1.684 (0.820–3.456) | 0.156 |
|  | NA | 63 | 47.6 | 1.412 (0.690–2.890) | 0.345 |
| Stage | IV | 142 | 45.8 | Reference |  |
|  | III | 38 | 52.6 | 1.068 (0.647–1.764) | 0.796 |
| Prior molecular targeted therapy | Present | 20 | 35.0 | Reference |  |
|  | None | 160 | 48.8 | 1.381 (0.637–2.994) | 0.414 |
| Prior radiotherapy | Thorax | 33 | 36.4 | Reference |  |
|  | Other than thorax | 19 | 52.6 | 1.679 (0.724–3.891) | 0.227 |
|  | None | 128 | 49.2 | 1.368 (0.738–2.539) | 0.320 |
| ICIs | Nivolumab | 99 | 45.5 | Reference |  |
|  | Atezolizumab | 11 | 18.2 | 0.330 (0.080–1.359) | 0.125 |
|  | Pembrolizumab | 70 | 54.3 | 1.142 (0.741–1.758) | 0.548 |
| Line of ICI therapy | 1st-line | 33 | 54.5 | 1.240 (0.685–2.244) | 0.477 |
|  | 2nd-line | 66 | 42.4 | Reference |  |
|  | ≥ 3rd-line | 81 | 48.1 | 1.143 (0.703–1.858) | 0.589 |
| WBC (/μL) | < 9000 | 146 | 43.8 | Reference |  |
|  | ≥ 9000 | 34 | 61.8 | 1.873 (1.141–3.075) | 0.013 |
| Platelets (/μL) | < 300000 | 123 | 45.5 | Reference |  |
|  | ≥ 300000 | 57 | 50.9 | 1.143 (0.730–1.791) | 0.559 |
| Neutrophils (/μL) | < 6000 | 132 | 47.7 | Reference |  |
|  | ≥ 6000 | 48 | 45.8 | 1.084 (0.667–1.762) | 0.745 |
| Eosinophils (/μL) | < 500 | 158 | 44.9 | Reference |  |
|  | ≥ 500 | 22 | 63.6 | 1.640 (0.924–2.914) | 0.091 |
| Basophils (/μL) | < 100 | 114 | 45.6 | Reference |  |
|  | ≥ 100 | 66 | 50.0 | 1.145 (0.739–1.773) | 0.545 |
| Monocytes (/μL) | < 600 | 116 | 45.7 | Reference |  |
|  | ≥ 600 | 64 | 50.0 | 1.232 (0.794–1.912) | 0.352 |
| Lymphocytes (/μL) | < 1500 | 103 | 37.9 | Reference |  |
|  | ≥ 1500 | 77 | 59.7 | 1.707 (1.114–2.617) | 0.014 |
| LDH (U/L) | < 230 | 112 | 46.4 | Reference |  |
|  | ≥ 230 | 68 | 48.5 | 1.140 (0.737–1.764) | 0.557 |
| Albumin (g/dL) | ≥ 4 | 50 | 40.0 | Reference |  |
|  | < 4 | 126 | 50.8 | 1.472 (0.889–2.439) | 0.133 |
|  | NA | 4 | 25.0 | 0.437 (0.059–3.265) | 0.420 |
| CRP (mg/dL) | < 1 | 96 | 41.7 | Reference |  |
|  | ≥ 1 | 84 | 53.6 | 1.469 (0.959–2.252) | 0.077 |
| NLR | < 5 | 129 | 48.8 | Reference |  |
|  | ≥ 5 | 51 | 43.1 | 1.031 (0.633–1.677) | 0.903 |
| LMR | < 3 | 97 | 42.3 | Reference |  |
|  | ≥ 3 | 83 | 53.0 | 1.192 (0.778–1.826) | 0.420 |
| PLR | ≥ 300 | 41 | 43.9 | Reference |  |
|  | < 300 | 139 | 48.2 | 1.035 (0.615–1.742) | 0.897 |
| irAEs, immune-related adverse events; HR, hazard ratio; CI, confidence interval; ECOG PS, Eastern Cooperative Oncology Group performance status; IIPs, idiopathic interstitial pneumonias; RIPF, radiation-induced pulmonary fibrosis; PE, pulmonary emphysema; ILD, interstitial lung disease; SCC, squamous cell carcinoma; EGFR, epidermal growth factor receptor; NA. not available; PD-L1, programmed cell death ligand-1; TPS, tumor proportion score; ICIs, immune checkpoint inhibitors; WBC, white blood cell; LDH, lactate dehydrogenase; CRP, C-reactive protein; NLR, neutrophil-to-lymphocyte ratio; LMR, lymphocyte-to-monocyte ratio; PLR, platelet-to-lymphocyte ratio. | | | | | |

| **Table S4** Univariate and multivariate analyses of ICI pneumonitis | | | | | |
| --- | --- | --- | --- | --- | --- |
| Parameter | | n | Pneumonitis  (%) | Univariate |  |
|  |  |  |  | HR (95% CI) | *P* value |
| Age (years) | ≥ 75 | 138 | 4.8 | Reference |  |
|  | < 75 | 42 | 18.1 | 3.991 (0.945–16.850) | 0.060 |
| Sex | Female | 40 | 10.0 | Reference |  |
|  | Male | 140 | 16.4 | 1.698 (0.587–4.909) | 0.329 |
| Smoking history | Non-smoker | 28 | 7.1 | Reference |  |
|  | Smoker | 152 | 16.4 | 2.549 (0.604–10.770) | 0.203 |
| ECOG PS | 0–1 | 163 | 14.7 | Reference |  |
|  | 2–3 | 17 | 17.6 | 1.787 (0.537–5.945) | 0.344 |
| Pre-existing respiratory disease | None | 61 | 6.6 | Reference |  |
|  | IIPs | 20 | 35.0 | 5.836 (1.708–19.940) | 0.005 |
|  | RIPF | 21 | 19.0 | 3.353 (0.839–13.410) | 0.087 |
|  | PE without ILD | 74 | 16.2 | 2.679 (0.864–8.309) | 0.088 |
|  | Others | 4 | 0.0 | < 0.001 (0–Inf) | 0.997 |
| Histologic type | Adenocarcinoma | 100 | 14.0 | Reference |  |
|  | SCC | 47 | 14.9 | 1.051 (0.424–2.605) | 0.915 |
|  | Others | 33 | 18.2 | 1.289 (0.495–3.356) | 0.603 |
| EGFR mutation | Positive | 21 | 0.0 | Reference |  |
|  | Negative | 130 | 17.7 | > 1000 (0.000–NA) | 0.997 |
|  | NA | 29 | 13.8 | > 1000 (0.000–NA) | 0.997 |
| PD-L1 TPS | < 1% | 49 | 24.0 | 4.186 (1.046–16.750) | 0.043 |
|  | 1–49% | 43 | 3.0 | Reference |  |
|  | ≥ 50% | 25 | 23.7 | 3.731 (1.052–13.230) | 0.042 |
|  | NA | 63 | 9.5 | 1.456 (0.364–5.822) | 0.595 |
| Stage | III | 38 | 15.8 | Reference |  |
|  | IV | 142 | 14.8 | 1.007 (0.406–2.496) | 0.988 |
| Prior molecular targeted therapy | Present | 20 | 0.0 | Reference |  |
|  | None | 160 | 16.9 | > 1000 (0.000–NA) | 0.997 |
| Prior radiotherapy | None | 128 | 14.1 | Reference |  |
|  | Thorax | 33 | 18.2 | 1.369 (0.543–3.448) | 0.506 |
|  | Other than thorax | 19 | 15.8 | 1.144 (0.337–3.885) | 0.829 |
| ICIs | Nivolumab | 70 | 14.1 | Reference |  |
|  | Atezolizumab | 99 | 9.1 | 0.603 (0.079–4.589) | 0.626 |
|  | Pembrolizumab | 11 | 17.1 | 1.129 (0.522–2.442) | 0.758 |
| Line of ICI therapy | 1st-line | 33 | 18.2 | 1.256 (0.447–3.535) | 0.665 |
|  | 2nd-line | 66 | 13.6 | Reference |  |
|  | ≥ 3rd-line | 81 | 14.8 | 1.049 (0.442–2.492) | 0.913 |
| WBC (/μL) | < 9000 | 146 | 12.3 | Reference |  |
|  | ≥ 9000 | 34 | 26.5 | 2.682 (1.201–5.986) | 0.016 |
| Platelets (/μL) | < 300000 | 123 | 14.6 | Reference |  |
|  | ≥ 300000 | 57 | 15.8 | 1.142 (0.513–2.543) | 0.745 |
| Neutrophils (/μL) | < 6000 | 132 | 13.6 | Reference |  |
|  | ≥ 6000 | 48 | 18.8 | 1.593 (0.714–3.553) | 0.255 |
| Eosinophils (/μL) | < 500 | 158 | 12.7 | Reference |  |
|  | ≥ 500 | 22 | 31.8 | 2.681 (1.133–6.343) | 0.025 |
| Basophils (/μL) | < 100 | 114 | 13.2 | Reference |  |
|  | ≥ 100 | 66 | 18.2 | 1.413 (0.661–3.019) | 0.373 |
| Monocytes (/μL) | < 600 | 116 | 8.6 | Reference |  |
|  | ≥ 600 | 64 | 26.6 | 3.655 (1.670–8.001) | 0.001 |
| Lymphocytes (/μL) | < 1500 | 103 | 11.7 | Reference |  |
|  | ≥ 1500 | 77 | 19.5 | 1.686 (0.789–3.604) | 0.178 |
| LDH (U/L) | < 230 | 112 | 15.2 | Reference |  |
|  | ≥ 230 | 68 | 14.7 | 1.066 (0.488–2.331) | 0.872 |
| Albumin (g/dL) | ≥ 4 | 50 | 6.0 | Reference |  |
|  | < 4 | 126 | 19.0 | 3.508 (1.056–11.660) | 0.041 |
|  | NA | 4 | 0.0 | < 0.001 (<0.001–Inf) | 0.997 |
| CRP (mg/dL) | < 1 | 96 | 7.3 | Reference |  |
|  | ≥ 1 | 84 | 23.8 | 3.814 (1.612–9.028) | 0.002 |
| NLR | < 5 | 129 | 14.0 | Reference |  |
|  | ≥ 5 | 51 | 17.6 | 1.394 (0.626–3.108) | 0.416 |
| LMR | ≥ 3 | 83 | 9.6 | Reference |  |
|  | < 3 | 97 | 19.6 | 2.256 (0.985–5.166) | 0.054 |
| PLR | < 300 | 139 | 12.9 | Reference |  |
|  | ≥ 300 | 41 | 22.0 | 1.843 (0.828–4.106) | 0.135 |
| ICI, immune checkpoint inhibitor; ICI-P, ICI pneumonitis; HR, hazard ratio; CI, confidence interval; ECOG PS, Eastern Cooperative Oncology Group performance status; IIPs, idiopathic interstitial pneumonias; RIPF, radiation-induced pulmonary fibrosis; PE, pulmonary emphysema; ILD, interstitial lung disease; SCC, squamous cell carcinoma; EGFR, epidermal growth factor receptor; NA, not available; PD-L1, programmed cell death ligand-1; TPS, tumor proportion score; WBC, white blood cell; LDH, lactate dehydrogenase; Inf, infinity; CRP, C-reactive protein; NLR, neutrophil-to-lymphocyte ratio; LMR, lymphocyte-to-monocyte ratio; PLR, platelet-to-lymphocyte ratio. | | | | | |
